# Large Corpora and Historical Syntax: Consequences for the Study of Morphosyntactic Diffusion in the History of Spanish

**DOI:** 10.3389/fpsyg.2019.00780

**Published:** 2019-04-17

**Authors:** Álvaro S. Octavio de Toledo y Huerta

**Affiliations:** Department of Spanish (Filología Española), Universidad Autónoma de Madrid, Madrid, Spain

**Keywords:** morphosyntactic change, diffusion, S-shaped curves, grammaticalization, dialectal variation, textual variation

## Abstract

Over the turn of the 21st century, the use of data from large electronic corpora has changed research on Spanish historical syntax, spurring interest in long-range evolutions, and the shape of the correspondent diachronic curves. However, general reflections on diffusion and the factors that drive and influence it are still pretty much lacking. In this paper, I reflect on the research possibilities laid open by the availability of such large masses of data, focusing particularly on new knowledge on syntactic change brought about by the study of low-frequency phenomena and of recessive changes, as well as on the exploration of changes conditioned by dialect contact, and textual traditions. I conclude with some remarks on the general typology of diffusion in syntactic change.

It is widely recognized that the field of historical morphosyntax in Spanish has been gaining ground in the last four decades to the extent that it now occupies a position of clear dominance in the area of diachronic linguistic research on that language (cf. Cano, 1991, 1995, 2000; Concepción Company Company, 2005; Girón, 2005, 2006; Kabatek, 2012, amongst others). This is reflected in the persistent dominance of work in this field as recorded in the proceedings of the conferences organized by the Association of Spanish Historical Linguistics (AHLE, *Asociación de Historia de la Lengua Española*^1^), a meeting held since 1987^2^. Looking at the contributions to the *Historical Morphology and Syntax* section in AHLE proceedings and taking only those in which primary data are used (i.e., data have not been merely collated from previously published sources), it is possible to group those contributions according to the time period studied, as shown in Table 1.

**Table 1:** Studies based on primary data collection included in the historical morphology and syntax section of the AHLE in the proceedings of the first seven conferences (1987–2006).

		Classical	Modern
		Spanish	Spanish
Congress (year of	Medieval	(16–17th	(18–19th	Holochronic
proceedings)	Spanish	centuries)	centuries)	studies
*I CIHLE* (1988)	25 (80,5%)	5 (16%)	0	1 (3%)
*II CIHLE* (1992)	38 (77,5%)	7 (14%)	1 (2,5%)	3 (6%)
*III CIHLE* (1996)	17 (77%)	0	1 (1%)	5 (22%)
*IV CIHLE* (1998)	22 (63%)	6 (17%)	0	7 (20%)
*V CIHLE* (2002)	21 (57%)	9 (24%)	1 (3%)	6 (16%)
*VI CIHLE* (2006)	25 (46%)	5 (9%)	2 (4%)	22 (41%)
*VII CIHLE* (2008)	18 (37,5%)	4 (8%)	4 (8,5%)	22 (46%)
Total (277)	166 (60%)	36 (13%)	9 (3%)	66 (24%)


From Table 1 two important conclusions are easily drawn: first, the body of work dedicated exclusively to the study of medieval Spanish has been diminishing at a steady rate; and secondly, in contrast, those works seeking to capture the complete historical evolution of the language (*holochronic* studies) have increased dramatically in frequency – the largest increase occurring around the time of the 6th Conference (Madrid, 2003). This parallel and complementary change in focus may have numerous causes, of which two are, in my opinion, clearly prevailing: a growing tendency to carry out (and, what is more, accept the validity of) studies dealing with very long timescales based on very few sources for the individual historical periods studied – these sources being considered representative due to their iconic literary or cultural status – and, above all, the increasing ease of access to large databases of digitized resources. A few years ago, Rolf Eberenz made a timely observation on both these trends, adding a warning concerning the implications of both:

We have at our disposal a body of texts, the majority of which are “literary,” from which we select two or three works for each century to extract the data that interests us […]. The digital corpus and the tools provided by information technology permit both statistical and linguistic analysis of increasing precision. However, the plethora of information stored on computer brings with it a certain danger, that is, the data tend to become a shapeless mass in which the differences between texts and within individual texts become lost from view [Eberenz, 2009: 189 (my translation, ÁOdT)].

The Diachronic Corpus of Spanish (CORDE^3^) is by far the digital corpus that has had most impact on the discipline, as Table 1 suggests, since the major leap upward in the trend for holochronic studies coincides with the granting of general access to this corpus at the beginning of this century. CORDE offers many pit-falls for the researcher, and not just those of the type which Eberenz outlines. It has been noted, for example, that there is a lack of philological quality in some of the texts available. There are also issues concerning how texts are dated (the date is fixed according to the original composition rather than the date of the much later manuscripts upon which the versions in the corpus are often based), the searchability of the database itself (an especially important consideration for syntactic searches), and the distribution of the texts by historical period (with some periods being far better represented than others)^4^. Despite these shortcomings, CORDE represents —as stated by Guillermo Rojo, its main promoter— an “instrumental revolution” (Rojo, 2012: 433–434) that has transformed the discipline of historical linguistics at its foundations, i.e., from the access to data itself. A strong indication of CORDE’s influence can be found in the 36 studies of morphosyntax in the most recent AHLE (Cádiz, 2012, proceedings from 2015), of which only a quarter (9/36) were completed without access to an electronic corpus, while over half (19/36) used data mined from CORDE^5^. In a branch of linguistics so dependent upon the bulk of data, this step change in available tools has brought with it a considerable reassessment of its objectives (cf. also Jenset and McGillivray, 2017). Within only 20 years, a large proportion of scholars in this area appear to have moved their focus from the detailed study of medieval morphosyntax (with very few excursions into other periods) to the tracing of and analysis of *holochronic trajectories* (evolutionary curves) of their chosen linguistic phenomena.

The exponential increase in available data and the transformation in form and substance that the exploitation of these data have instigated thus constitute one of the most substantial changes to the field of historical morphosyntax in Spanish in recent years. Over the rest of this article, I will present an overview of the benefits of this abundance of data – deliberately leaving aside the disadvantages^6^. I will conventionally divide these advantages into two categories (although in practice they frequently overlap), namely *quantitative* ones, such as the ability to explore very low frequency phenomena or the establishment of more precise evolutionary trends, and *qualitative* ones, such as the opportunity to observe correlations between certain well defined trends or, supported by better data, further explore the patterns of change in a particular phenomenon in terms of its diatopic variation or its variation throughout different textual classes (*discourse traditions*), for instance.

As I will show, a resource like CORDE allows us to study constructions which appear with infinitesimally small frequency such as, for example, the schema in which an infinitive is placed before the auxiliary verb *tener* “to hold, to possess,” either with an intermediate object clitic (*le* in example 1c) or without it (examples 1a and b), and either with a linking preposition *de* “of”next to the infinitive (example 1a) or without the preposition (examples 1b and c)^7^.

(1) a. *non solamente a su coamante* de dar tyene, *mas a otras çyento ha de contentar*
  not only to his lover did he *have to give*, but another hundred were there to satisfy
  ([Alfonso Martínez de Toledo, *Corbacho*, 1438 (ms. de 1466)].
  b. *¿quién pensar pudiera que así las fuerças de mi propósito* enflaquecer tenían?
  who would have thought that this is how the forces of my purpose *had to get thinner*?
  (*Diego de San Pedro, Arnalte, 1446–1447, 124*).
  c. *no puedo más*, seguirle tengo; *somos de un mismo lugar*
  I can do no more, I *have to follow him*, we come from the same place
  (*Quijote*, II, 33, 906).

The evolution of the use of this syntactic schema is shown in Figure 1 (cf. Octavio de Toledo y Huerta, 2016b) from which we can determine two main points: firstly, whilst this construction was in use (that is, from the middle of the 15th Century to around 1650), it saw sustained growth: see the thickest line on the graph which represents, as a percentage, the proportion with which all variants of the schema appear in a particular time period out of the total number of cases observed^8^; and secondly, between the end of the fourteen hundreds and the middle of the 16th century there was a rapid increase in the use of variants involving no linking preposition and of those including a clitic (see the fine, continuous line and the large dashed line, respectively), thus becoming increasingly analogs to the so-called “analytic future” of the type *cantarlo he* “I will sing it,” literally “to sing it (I) have,” which obligatorily includes a clitic and has no linking preposition. Furthermore, and in concert with these formal changes, a distributional change also took place as the construction came to be more often found toward the beginning of main clauses, further converging with “analytic futures,” which are almost exclusively found in that position^9^.

**FIGURE 1:**
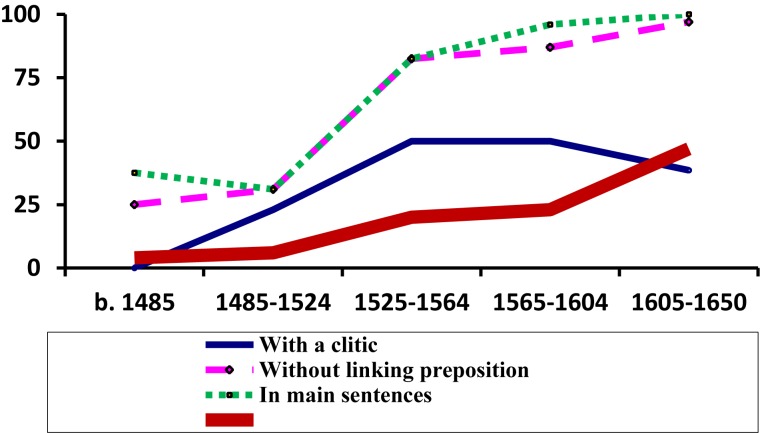
Percentage values (for each time period) for different schemas and global, relative frequency of all schemas of the Infinitive + *tener* construction.

In early Classical Spanish, *haber* “to have” had to a large extent lost its possessive meaning and was being lexically replaced by *tener* “to hold, to possess.” This substitution had reached modal deontic periprases such as *haber de* + INF, which was losing ground to *tener de* + INF (cf. Garachana, 2011). Since also the construction in (1), with *tener*, increasingly converged with the syntactic behavior of the already available “analytic future” with *haber*, it seems reasonable to assume that speakers still had some way of recognizing an autonomous auxiliary-like element at the end of *cantarlo he* as suggested by Anipa (2000). This is in line with the idea – already formulated by Nebrija, no less – that the so-called “analytic future” is not a strange form of “interrupted future” with a disrupting clitic, but rather a periphrasis similar to those formed using other modal verbs, which until 1660 are also commonly “fronted” (i.e., placed before the infinitive) and can be accompanied by clitics, as in the case of *decirlo +* {*debo/puedo/quiero*} “say-it (I) {must/can/want}”). After 1600, the whole set of constructions using the infinitive followed by an inflected modal verb practically disappears (cf. Octavio de Toledo y Huerta, 2015a). This mass extinction includes those schemas involving the auxiliary verb *tener*; yet, as Figure 1 demonstrates, at the moment of its disappearance this construction appears to be gaining frequency rather than showing a decline in usage. In this way, the loss of sequences containing INF (+ clitic) + modal verb (including the “analytic future” *cantarlo he*) seems to have occurred almost catastrophically (cf. Bernárdez, 1994; López García, 1996, 2011), possibly because the motivations behind this decline do not lie in the gradual loss of enclisis in V1 positions, as has been traditionally argued, but rather in a relatively rapid change in sentence structure, specifically, the information-structural properties of the leftmost (non-peripheral) edge of a (main) sentence, a position from which, from the end of the 16th and the middle of the 17th centuries, non-quantified phrases bearing focus seem to have been excluded (cf. Mackenzie, 2010; Sitaridou, 2011; Sitaridou and Eide, 2014; Batllori and Hernanz, 2015; Batllori, 2016). Be it as it may, it is not without interest – and this is what I would like to underline here – that some of the strongest indications of the periphrastic character of *cantarlo he* (and hence of its close ties to a wide group of schemas all corresponding to a common basic configuration and disappearing simultaneously) come directly from the very rare group of schemas illustrated in (1), the systematic study of which would not have been possible without the resources of an immense digitized corpus. Thus, the analysis of low frequency phenomena can have an impact that reaches far beyond the merely descriptive and, on occasion, may go on to open doors to the formulation of new hypotheses concerning the evolution of much larger groups of constructions.

Looking more widely, the interest in poorly documented changes will, I believe, also change the way in which we collect data, pushing us to search further afield than our conventional corpora (in this context, conventional is meant in the sense of controlled and closed, with a finite number – however large that might be – of representative sources that have been selected according to certain criteria). It will often be the case that only a handful of data on a particular construction or syntactic schema can be extracted from resources such as CORDE (or its recent upgrade, CDH), the Corpus of Spanish (*Corpus del Españo*l), CODEA+, CORDIAM or the search engine provided by the Biblioteca Virtual Miguel de Cervantes (BVMC), to mention only the major corpora available that allow exploring not only Medieval Spanish but also later periods^10^. In such situations, researchers may become increasingly inclined to search through databases that are open – i.e., constantly growing in number – and not mediated, in the sense that the works they hold are not selected according to philological criteria, but if anything, rather on bibliological grounds, and contain versions in substantially original format (untouched by a modern editor), like Google Books^11^. It is possible to find on this platform, for example, dozens of examples which confirm the exclusively eastern^12^ character of the prepositional use of *bajo +* NP (*bajo la cama*, “under the bed”) in Pre-classical and Classical Spanish [example (2); cf. Octavio de Toledo y Huerta, 2015b], which shows, in turn, that it is not trivial but urgent to increase the efforts to extend dialectal studies on Iberian ground to include all available printed sources and into the modern era.

(2) *Mi marido está* baxo *la cama*
  My husband is *under* the bed
  (*Exemplario contra los engaños y peligros del mundo*, Zaragoza, 1493).
  *Estavan* baxo *el árbol confundidos hombres y brutos*
  They were *under* the tree, men and beasts all together
  (*Baltasar Gracián, Criticón, II, 205*).

On the other hand, in the wake of the pioneering work of scholars such as Morala (2002) and García de Paredes (2011), there has been a growth in studies looking at low frequency phenomena by using the broadest and most unrestricted corpus available, i.e., the Internet, a particularly useful move whenever a phenomenon’s low incidence in standard corpora may be connected to its evident diatopic or diaphasic markedness, which will tend to preclude its appearance in corpora largely dominated by works conforming to the linguistic standards of highly elaborated literature. The Internet, of course, yields extremely multifarious data, the correct discrimination and contextualization of which requires huge philological effort. However, it is not difficult to find pearls in this deep electronic ocean where the consultation of conventional corpora only offers a glimpse of a tantalizing phenomenon. For instance, whilst CORDE finds just one example of the quantifier *algotro* “some other (thing or person)” (3), by using the geographical associations provided by data from Google^13^, we find that it is a dialectal feature of Extremadura (whence Felipe Trigo came) and western La Mancha (rather than of Andalucia, *pace* the Real Academia Española’s dictionary).

(3) *Unas cosa las vide yo mesmo, por mis ojo*; algotras *de endenantes, y de las que hición los tres en la ermita con aquellas probe*
  Some things I saw myself, with my own eyes; *some others* a while ago, and what the three of them did in the chapel with those poor women
  (Felipe Trigo, *Jarrapellejos*, 1914).

Turning (just once) to the lexicon instead of syntax, the diminutive form *mengajo* (originally “rag,” then also “little child”) is assigned a Murcian origin in the old and venerable *Diccionario de Autoridades* (1726–1739), but neither CORDE/CDH nor their contemporary counterparts, CREA/CORPES XXI, contribute one single example; its persistence as a south-eastern hallmark is confirmed via Google searches, which also reveal the contemporary spread of this term into the eastern regions of La Mancha. Map 1 shows the prevalence of *algotro* (toward the west, circles, and triangles) and *mengajo* (toward the southeast, ovals, and squares) as found using Google (the basic parameters, restrictions, and limitations of the searches have been detailed elsewhere: cf. Octavio de Toledo y Huerta, 2016c).

**MAP 1:**
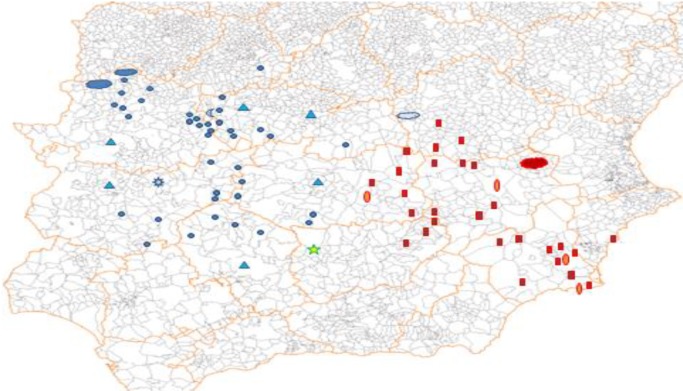
Spanish results that can be localized geographically from Google searches for *algotro* “some other” (circles and triangles) and *mengajo* “rag, little child” (ovals and squares), *apud* Octavio de Toledo y Huerta, 2016c.

These two examples suffice, in my opinion, as evidence of the degree to which these new data sources will facilitate our access to linguistic phenomena difficult to trace until now. This is likely to modify our views on phenomena still disregarded as residual or marginal, thus shining a light on the evolution of broader processes: the western distribution of *algotro*, for instance, perfectly coincides with that of the demonstratives *estotro*/*esotro* “this other/that other” (cf. Octavio de Toledo y Huerta, 2018a), similarly formed on *otro* “another,” and at the same time with the western origins of *alguien* “somebody” (Malkiel, 1948; Pato, 2009), another indefinite quantifier which uses the prefix *alg-*, as well as with the presence in Galician and Portuguese of other indefinite lexemes containing *alg-*, such as *algures* “somewhere,” and on the other hand with the historical scarcity or absence of examples of Sp. And Port. *algo* “something” toward the eastern territories (note that standard Catalan displays but one *alg*- form, as present in *algú*/*alguna cosa* “someone/something,” cf. Sp. *algún*, port *algum*). Thus, both a full-fledged system of indefinites with *alg*- and the formation of pronominal compounds with *otro* appear to be western features, as confirmed by the decidedly western status of *algotro*, where both the *alg*- radical and the compounding procedure converge.

With respect to establishing more precise evolutionary curves, it is not my intention here to discuss technical improvements, such as the possibilities for multifactorial analysis brought along with the programming language R (cf. a.o. Gries, 2009; Bivand et al., 2013: 151–166, Arnold and Tilton, 2015 or Levshina, 2015) or refinements in quantitative approaches, particularly in the area of inferential statistics [cf., with particular reference to the history of Spanish, the splendid book by Rosemeyer (2014)]. I will instead focus on a complementary aspect of this endeavor which may have greater theoretical importance. Works such as Rosemeyer (2014) analysis of the extinction in Spanish of the non-passive constructions with *ser* “to be” and a past participle, Marco and Marín (2015) remarks on the expansion of *estar* “to stand, to stay, to be” + participle or the already classic study by Rodríguez Molina (2004) about the diffusion of *haber* “to have” + participle all clearly show, through a wealth of data, that the progression or regression of these auxiliaries is a function of their progressive adoption or rejection by specific groups of predicates. For example, participles that convey the continuity or emergence of an event, such as *permanecer* “to remain” or *suceder* “to happen,” lose their ability to combine with *ser* at an earlier stage than others (e.g., participles indicating change of state); the participles of verbs of transfer, like *dar* “to give,” become associated with auxiliary *haber* “to have” sooner and more frequently than with other verbs; and the huge increase in the use of *estar* + participle in Classical Spanish (roughly, the 16th and 17th centuries) largely coincides with its adoption by psychological predicates involving an experiencer, such as *preocupar* “to worry.” In all these cases, the observed diffusion or regression of the constructions follows a logistic curve, or S-curve (cf. Kroch, 1989; by means of example, Figure 2 shows the decline in usage of *ser* + resultative participle), and it does seem that such a curve is indeed characteristic for syntactic change that involves a form of diffusion (whether progress or decline) mediated by lexical permeability.

**FIGURE 2:**
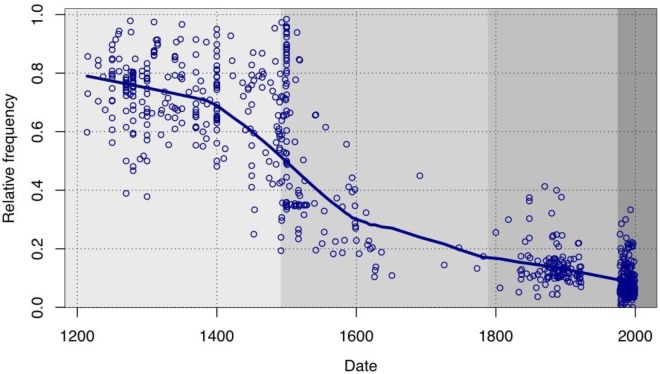
Evolution of the relative frequency of the construction *ser* “to be” + resultative participle (from Sánchez Marco, 2012: 99, reproduced by kind permission of the author).

It might certainly be the case that the S-curve is actually the only function which can properly model the diffusion of syntactic change, as asserted by Blythe and Croft (2012) (cf. also Nevalainen, 2015 or Feltgen et al., 2017)^14^, who blame the peculiar curvature on how a community of speakers evaluate a linguistic variant in terms of its prestige within their social group, regardless of the structure of said social group.

The overall structure of the trajectory of a language change [is] an S-curve, no matter how it [is] propagated through grammatical contexts, words, speakers, texts, geographical regions, or social classes. This overall trajectory appears to be determined by differential weighting of variants (replicator selection) (Blythe and Croft, 2012: 294).

Besides lexical diffusion (i.e., “through words”), these authors explicitly mention a further mode of diffusion via syntactic patterns (i.e., “through grammatical contexts”). The latter would appear to be in action in the expansion of the shorter variant *hemos* at the expense of the older form *habemos* (both meaning “we have”), which unfolds along another sinuous S-curve principally during the Classical Spanish period (Figure 3; cf. Bustos and Moreno, 1992; Rodríguez Molina, 2012). The process clearly appears to have been guided by the progressive expansion of the variant *hemos* out of its natural environment of origin in Old Castilian (as part of the “analytic future,” i.e., the INF + Clitic + AUX (*have*) construction: *cantarlo hemos* “we will sing it”) to the formally and, most importantly, semantically related deontic periphrasis *haber de* + INF “have to + INF,” and there from, in successive waves, to the perfect tenses with *haber* + Past Participle “have PP,” less related to the source constructions both in form and meaning. This is suggested by the data in Table 2, the result of an exhaustive search in CORDE for 1500–1530, the period when the shorter variant *hemos* saw its initial boom. In global terms, Table 2 shows that *hemos* and *habemos* occur with similar frequencies (50% of the total for each form) within the corpus; however, the variant *hemos* shows the greatest affiliation to *haber de* + INF (70% of examples of this periphrasis use the shorter variant, while it is the preferred form in only 41% of cases of *haber* + PP), whereas the variant *habemos* is shown to be largely preferred in non-auxiliary contexts (where *haber* is used as a verb of possession), clearly more distant from the periphrastic futurate construction where short *hemos* originated.

**FIGURE 3:**
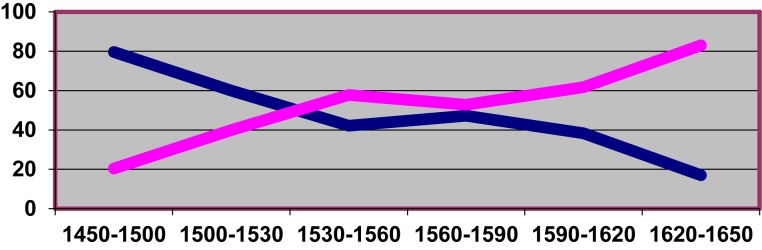
Change in relative frequency (expressed as a percentage) for *hemos* (rising curve) vs. *habemos* (descending curve) throughout Classical Spanish.

**Table 2:** Frequencies of use (total instances and as a percentage) of *hemos* and *habemos* in different syntactic contexts.

	HEMOS	HABEMOS	TOTAL
Analytical future	165 (100%)	0	165
*Haber de* + INF	358 (70%)	154	512
*Haber* + PP	594 (41%)	853	1.447
Possessive use	32	153 (83%)	185
Σ	1.149 (50%)	1.160 (50%)	**2.309**


*Data taken from the CORDE database for the period 1500–1530.*

Rosemeyer (2016), for example, also observes an expansion through syntactic contexts in the competition between *ser* + PP and *haber*+ PP with reflexive predicates^15^. As De Smet (2012, 2013) suggests, such processes evidence the importance of extension via similarities between related syntactic contexts during the enactment of a change taking place over the medium or long term. However, of most interest here is that it is not always certain that this type of syntactic extension will behave in exactly the same way as lexical extension in terms of its diffusion. In fact, the curve shown on Figure 3 does not at first glance show the typical characteristics of an S-curve, beginning and ending with a shallow gradient while showing a much higher rate of change in its intermediate range. On the contrary, its central region suggests a period of relative stability after an initial surge and it finishes on a similarly steep trajectory (although, of course, both a slow inception before 1450 and a slow tapering-off after 1650 can be reasonably assumed)^16^. The reason for this may lie in the fact that the S-curve is a natural feature of lexical diffusion: initially, very few words will adopt a change; then, during the intermediate phase, given that the semantic connections between lexical elements form a complex network, groups of interconnected words will add together in a cascade with a cumulative, snow-balling effect; finally, only a few isolated areas of resistance remain, which explains the slow trailing off of the last phase. However, purely *syntactic context expansion* (cf. Himmelmann, 2004: 32–33) may follow a more irregular pattern: at some point the variant may come into use in several contexts simultaneously (or successively, but with very short time intervals between each), hence expand at a very high rate. However, once all the available areas of use have been accessed, its progress may stagnate as, in contrast to lexical diffusion, it does not receive an impulse from a sustained adoption on the part of a growing paradigm class (*host-class expansion*; cf. again Himmelmann, 2004: 32–33). The existence of a final stage of accelerated *mutation* – i.e., the abandonment or diasystematic isolation of one of the competing variants, as described by Coseriu (1983: 55) – is in all probability the result of a (half-)conscious, socially motivated bias on the part of speakers. Certainly, Blythe and Croft (2012) proposal refers not only to “pure” S-curves but also to any course of development compatible with such curves (Blythe and Croft, 2012: 293): the trajectory in Figure 3 could be seen as a two-staged S-curve made up of two successive S-curves, the second of which includes a significant phase of initial delay or “latency” (Feltgen et al., 2017)^17^. Such irregularities in the shape of S-curves, however, could be indicative of a differential intervention of endogenous vs. exogenous factors of change (cf. Ghanbarnejad et al., 2014), or of lexical vs. syntactic extension, or both. In any case, the study of further trajectories corresponding to other examples of syntactic extension should help narrow down the extent to which the observed differences can be generalized to a wider group of linguistic developments.

The establishment of an ever increasing set of frequency curves of sufficient precision invites comparison between them, an exercise that might uncover correlations poorly studied until recently. The GRADIA project, to which I belong^18^, has investigated the development of a wide group of modal periphrases, as shown in Figure 4. Interestingly, the increased use of *tener que*+ INF “to have to,” a periphrasis traditionally blamed for the regression of *haber de*+ INF, does not seem to directly prejudice the use of this construction until the dawn of the 20th century, which can be taken as an indication that, at first, these two constructions did not compete excessively to express the same values (*tener que*+ INF emerges as a clearly obligation periphrasis: cf. Garachana, 2016). On the other hand, both the increase and the decline in usage of *haber de*+ INF “have to” are inversely related, from 1500 onward, to the curves showing the use of *deber (de)*+ INF “must, ought to,” a fact which suggests that *haber de*+ INF became engaged in Classical Spanish in a competition to express not only deontic, but also epistemic values, since *deber (de)* + INF could convey both (cf. Garachana and Hernández Díaz, 2017).

**FIGURE 4:**
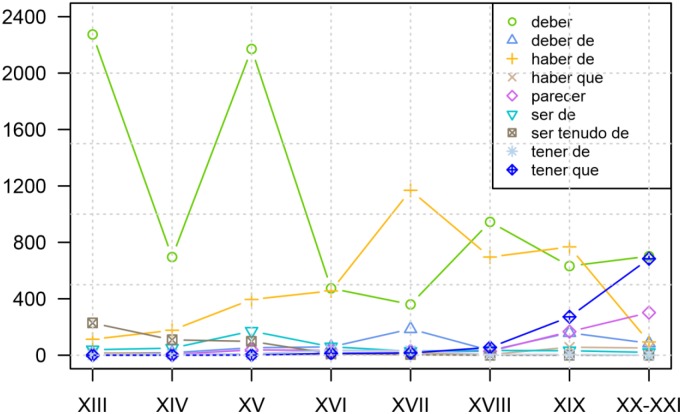
Evolution of the relative frequencies (per million words) of nine Spanish modal constructions with an infinitive, all of them deontic except with *parecer* “to seem” (elaboration by Malte Rosemeyer using data from various members of the GRADIA project: reproduced by kind permission of the author). The data were recovered from the GRADIA corpus: http://gradiadiacronia.wixsite.com/gradia/corpus-gradia.

Thus, Figure 4 helps us to understand the importance of co-evolution in groups of constructions that are similar both in form and meaning: a specific trajectory might be accelerated (or slowed down) by the appearance of others within its local variational environment or “envelope of variation,” that is, its constructional network (for analogical effects of attraction and differentiation within such networks, cf. now De Smet et al., 2018). The effects of co-evolution are also felt in the case of the periphrasis with fronted infinitive, for example, *cantar(lo) tengo* (see Figure 1 above): the curve shows how the presence of clitics within the schema grows significantly until the middle of the 16th century, which, as already stated, clearly indicates a convergence with the “analytic future” *cantarlo he*, a construction in which the clitic is obligatory. However, this tendency never reaches completion; instead, the curve levels off and even shows a clear decline in the 17th century, most probably due to the fact that the construction with *tener* departs from the model with *haber* (which was receding at great speed under the competence of the “synthetic” solution *cantarelo* “I will sing it”) and becomes attracted to analogous sequences using the auxiliaries *deber* “must, ought to,” *poder* “can, be able to” and *querer* “want to,” in which the clitic can be used but is not obligatory (Octavio de Toledo y Huerta, 2016b). In any case, what is important here is that the formulation of these hypotheses emerges directly from the comparison of evolutionary paths, whether they describe the coevolution of a whole network (Figure 4) or the syntactic properties of a single given phenomenon (Figure 1). Without observing these frequency curves, it would have been far more difficult for scholars to implement these new possibilities of analysis.

On the other hand, the trajectory of *haber de* + INF “to have to” (Figure 4) makes us wonder whether phenomena that become recessive at a certain point do always follow a descending S-curve even during this phase of recession (cf. e.g., Figure 2). Blythe and Croft (2012) do not deal with this type of change:

There are […] changes in our survey that appear to stop and go in reverse. These may be interpreted as changes following an S-curve trajectory that are then interrupted; we do not analyze such changes here (Blythe and Croft, 2012: 279).

Thus, like many other scholars, Blythe and Croft (2012) concentrate only on changes resulting in successful diffusion. The tendency to focus purely on the ascendant phase of certain changes is very common, for example, with remarks on the relationship between grammaticalization and frequency:

As long as frequency is on the rise, changes will move in a consistent direction […]. When a grammaticalization construction ceases to rise in frequency, various things happen, but none of them is the precise reverse of the process (Bybee, 2011: 77).

The increase in frequency, then, is a symptom of grammaticalization, but we are still none the wiser as to how to interpret the downturn in terms of that very same model of morphosyntactic change. Given that there is no need to assume that the changes which become generalized [i.e., those that reach Coseriu (1983) stage of *mutation*] will be more abundant than those that fail to develop part way through their trajectory, it seems obvious that the studies of grammaticalization have still not managed to produce an unbiased model of diffusion, that is to say, one not restricted to the time period over which the grammaticalized element or schema is seen to expand^19^.

However, not only the recessive phases create doubts about the generalizability of S-curves^20^. The expansion in use of the article before subordinate clauses headed by *que* “that” (Figure 5; cf. Lapesa, 1984; Herrero, 2013; Octavio de Toledo y Huerta, 2014b) shows a pattern of diffusion that is difficult to fit to a function of this type. This phenomenon’s explosive surge rather conforms to an *exponential* curve, with a prolonged and shallow initial curve followed by a brisk upturn and without a third phase of moderate growth (note that the phenomenon becomes regressive after reaching its maximum value, gently falling back in compliance to the S-curve pattern).

**FIGURE 5:**
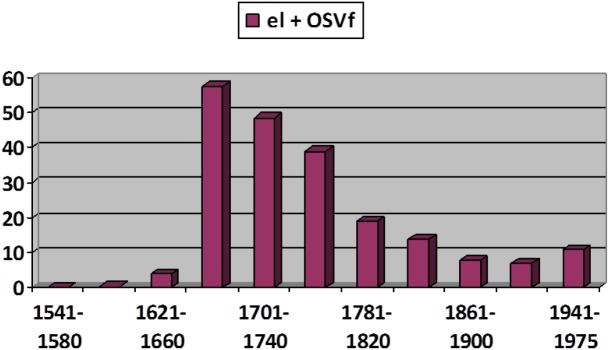
Diffusion of the use of the article (*el* “the.M.SG”) before clauses introduced by *que* “that” presented as a weighted frequency by time period. Data from CORDE (Octavio de Toledo y Huerta, 2014b).

Blythe and Croft (2012) explicitly disregard the existence of such trajectories, at least in the case of competing constructions of the kind envisaged by Kroch (1989) and themselves:

To our knowledge there are no clearly documented cases of a change going toward completion that follows […] an exponential curve (either slow start with a rapid completion and no tapering off, or an immediate rapid increase followed by a slow completion rate) (Blythe and Croft, 2012: 280).

But the construction in Figure 5 emerged as an alternative to other complementizer schemas without displacing any of them. It is worth noting here that we are dealing with a rather uncommon kind of curve, its main interest being that it invites reflection on whether this form of diffusion results from some special circumstances. In my opinion, the answer could be yes. The phenomenon in Figure 5 is best explained as an extension in the use of the article as a syntactic marker from a similar and pre-existent construction in which the article precedes an infinitive clause (where the infinitive exhibits a clear verbal value: cf. Torres, 2009). As shown in Figure 6, the “contagion” of the article to subordinate clauses introduced by *que* “that” occurred when its use with the infinitive clause was at its height (indicated with the lightest colored bar on Figure 6); when this schema enters its decline, the derived construction with *el* + *que* also decays. The growth of the schema in Figure 5 can be thus seen to rely on the success of another semantically similar schema serving as a *supporting construction* (cf. De Smet and Fischer, 2017). The phenomenon is by no means unique: it can be found in similar examples, such as the semantic extension of *sino es* “if not, but” from an exceptive meaning (*all were tall* ***but*** *Paul*) to becoming a corrective adversative linking sequence (*Paul was not tall*, ***but*** *short*: cf. Octavio de Toledo y Huerta, 2008). As Figure 7 shows, the adversative value starts to flourish quite abruptly at the same time as its exceptive use also reaches its apex and subsequently dies away, following an S pattern, at the same rate as the parent schema.

**FIGURE 6:**
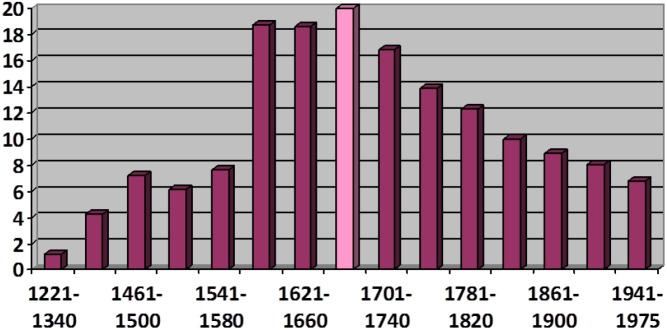
Article placed before infinitive clauses (data from CORDE, infinitives beginning with *a*- and *r*-).

**FIGURE 7:**
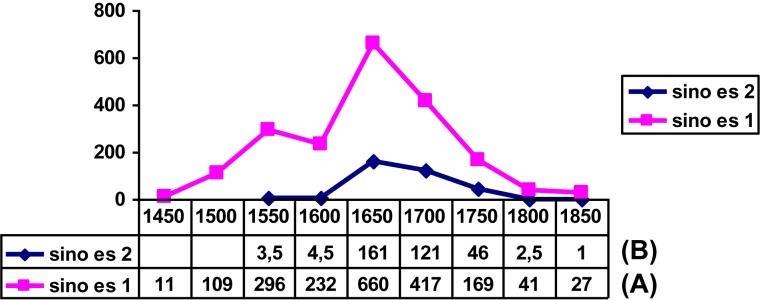
Evolution of weighted frequencies for *sino es* “if not, but”: **(A)** with an exceptive meaning (*sino es* 1: *no se casan sino es con permiso*, “they won’t marry but with permission,” and **(B)** with corrective adversative meaning (*sino es* 2: *no son pobres sino es ricos*, “they are not poor but rich”). Data from CORDE.

We might be confronted here with a specific form of diffusion that could be termed as “parasitic,” given the dependence of the derived construction upon the schema on which it is based. This type of extension to new semantic values or syntactic schemas appears to be typical of secondary grammaticalization (that which affects elements or sequences that already have a grammatical value; cf. especially Norde, 2012; Breban, 2014; Killie, 2015) and could produce very abruptly raising curves, which would thus be symptomatic of the mode of expansion and ensuing regression that Haspelmath (2004) terms *retraction*, i.e., the appearance and subsequent elimination of a function – in the sense of a new form-meaning pairing – toward the end of a grammaticalization chain (for the characteristic structures of such chains, cf. Heine, 1992). In all events, the formulation of this hypothesis, which surely needs further proof, is once again made possible by the observation of correlations between curves describing the trajectories of related phenomena.

The comparison of cognate trajectories is the strategy behind Postma (2010) claim that a low-frequency, aborted change can still fuel other changes with greater impact. For instance, the superposition of the curve showing the usage of the definite article before Wh-questions and that showing the use of the article before a subordinate clause introduced by *que* “that” (Figure 8) confirms [Lapesa (1984): 542–543] suggestion that the former schema, although short-lived and never too frequent, could stimulate the expansion of the latter (cf. Octavio de Toledo y Huerta, 2014b). Moreover, the rise of these clauses where an article precedes a complementizer *que* (*el* + *que*) could have buttressed, (according to Girón, 2004) the emergence of the homophonic sequence *el que* “which,” a newly grammaticalized compound relative pronoun formed with the article and a relative *que* (Figure 9). This possibility, however likely, naturally puts forth an additional question about the role of the replication of sequences already familiar to the speaker – i.e., sequences that are part of his competence and can serve as model for the processing and production of new sequences– as a triggering mechanism of grammatical change (*entrenchment via priming*: cf. e.g., Szmrecsanyi, 2005; Jaeger and Rosenbach, 2008; Schmid, 2016; for some other historical phenomena in Spanish, cf. Torres, 2015; Mackenzie, 2017; Rosemeyer and Schwenter, 2017 in print).

**FIGURE 8:**
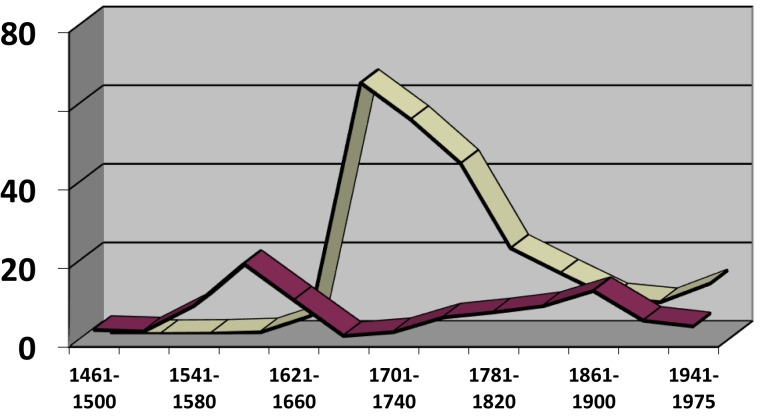
Weighted frequency curves showing the constructions ART+Wh (*me explicó el cómo lo hacían*, “he explained ART how it was done”: lower curve) and ART+C (*te agradezco el que vengas*, “I am grateful to you ART that you came”: curve with peak frequency in 18th Century). Data from CORDE.

**FIGURE 9:**
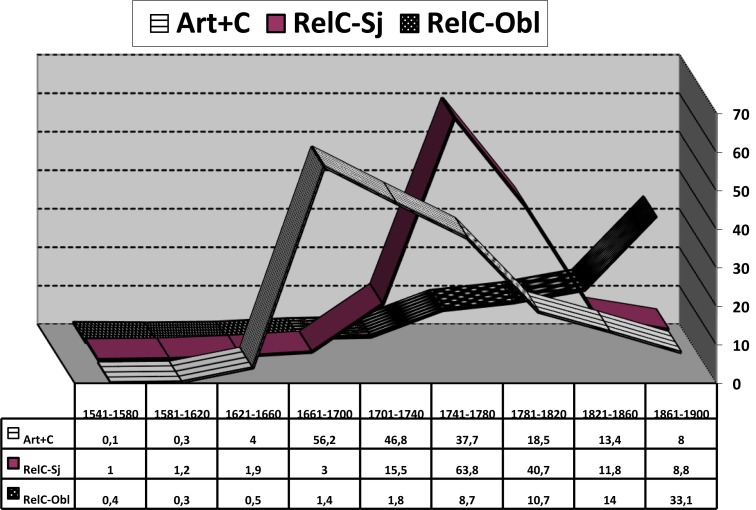
Weighted frequency curves of ART+C (striped band), non-oblique (subject/object) compound relative pronouns (solid band), and oblique relative pronouns (band with light dots on dark background). Data from CORDE.

Finally, with regards to the opportunity for improving our diasystemic (or variational) characterization of linguistic changes^21^, large corpora allow us to, once again, make important progress in little time. Thus, if Rodríguez Molina (2012) was able to demonstrate, through the use of a substantial corpus which he painstakingly gathered himself, that the early (i.e., before 1450) reduction in the use of the longer variant *habemos* in favor of shorter form *hemos* in compound tenses is an eastern phenomenon (Map 2A), the large Ibero-Romance corpora now available online allow us to establish in a few minutes that this decline was also early toward the west (in the possessive uses and in the modal periphrasis with the infinitive, since compound tenses are not used in these varieties of Spanish: Map 2B), and that the restriction of shorter form *hemos* to analytic futures until the middle of the fourteen hundreds is, therefore, a phenomenon unique to central Castilian Spanish.

**MAP 2:**
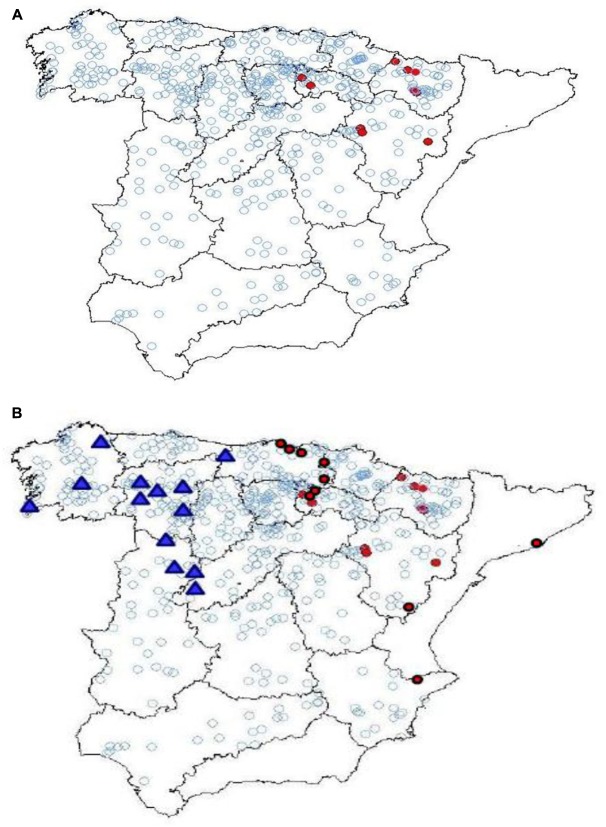
**(A,B)** Points where the shorter form *hemos* is found before 1450 according to Rodríguez Molina (2012) (top) and additional points established through consultation of the online corpora CORDE, CODEA+, TMILG, and CICA (bottom: western data is indicated with triangles).

Now, the study of how a change is adopted across discursive traditions is not only a methodological requirement of any investigation that seeks to take a non-trivial historical perspective^22^, but it can also provide the key for explaining the propagation of a given phenomenon. Such is the case with the schema in which the negative qualifier *nada* “nothing” is placed before the main verb, as in *nada sé* “nothing I know,” rather than the more common negative agreement construction, *no sé nada* “NEG I know nothing.” This schema expands and declines in use in accordance with the degree of acceptance of a syntactic rule imported into cultivated Spanish prose from the written tradition and grammar of Latin – a language with no negative agreement and typically preverbal negative quantifiers (*nihil scio* “Nothing I know”). As discussed elsewhere (Octavio de Toledo y Huerta, 2014a), the adoption of this rule can be easily tracked from its initial success amongst the first Spanish humanists in the fourteen hundreds, through highly elaborated works in a variety of genres, until the Romantic era, where, as part of a rejection of the classical rhetorical paradigm, the schema starts to fall into disuse, a trend which has continued to this day (Figure 10). This phenomenon illustrates the qualitative and quantitative gain derived from careful study of the discourse-traditional characteristics displayed by linguistic items and constructions (cf. the German adjective *diskustraditionell*: Kabatek, 2015; Winter-Froemel et al., 2015; Varga, 2017; Octavio de Toledo y Huerta, 2018b), which more often than not signals a unique trajectory for each phenomenon throughout its recorded textual history^23^. This endeavor, that departs from the itineraries traced by the phenomena themselves, seems more profitable to the interests of historical morphosyntax than that of establishing what might be called a taxonomy of discursive traditions and attempting to ascribe to them certain (allegedly) characteristic morphosyntactic features.

**FIGURE 10:**
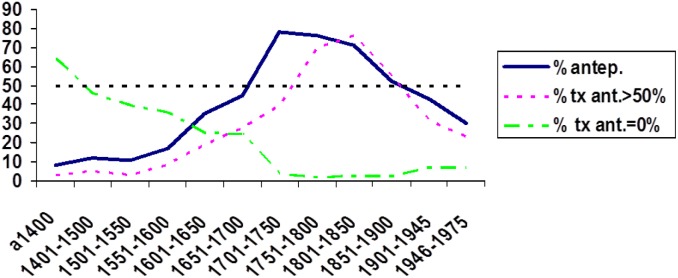
Proportion of appearances of the schema *nada sé* (as a percentage of the sum of these cases and those of the type *no sé nada*: % antep.), proportion of texts where *nada* “nothing” appears before the verb in more than 50% of cases (% tx ant. >50%) and proportion of texts where there are no cases of *nada* “nothing” before the verb (% ant. = 0%). Data from CORDE.

I am well aware that I have presented more dilemmas and possibilities than solutions throughout this paper. However, I want to finish by introducing yet another challenge: perhaps the time is ripe already to start building the foundations for a classification of linguistic diffusion, distinguishing, for example, the regular S-curves, which one expects in the case of diffusion linked to competing pairs, from other curves that are possibly less uniform in their final stages such as those associated with diffusion via syntactic extension, or from ascending phases that do not seem to follow a logistic curve, and considering not simply the dynamics of these phases of rampant ascent but also regressive phases, which have been rather neglected until now. Indeed, we must attempt to profile the specific forms of linguistic diffusion that fit specific manifestations of linguistic change, as in the class of propagation we have termed “parasitic,” whose relationship with expansion and retraction at the extremes of grammaticalization chains places it in opposition to, for example, forms of propagation in which the appearance of a new schema diffuses at the expense of similar forms or constructions that preceded it, which it ultimately replaces. This is exactly what happened in the evolution of the temporal conjunction *ínterin* “while,” which from its first appearance replaces, at an ever-increasing rate, its competitor *ínterin que* “meanwhile that,” which in turn had previously ousted the equivalent conjunctive phrases *en (el) ínterin que* “in (the) meantime that” (Figure 11; cf. Octavio de Toledo y Huerta, 2007). This form of diffusion could maybe be dubbed “phagocytic” or “cannibalistic”. In this way, it becomes possible to start a catalog of the specific forms of change corresponding to particular forms of linguistic diffusion^24^.

**FIGURE 11:**
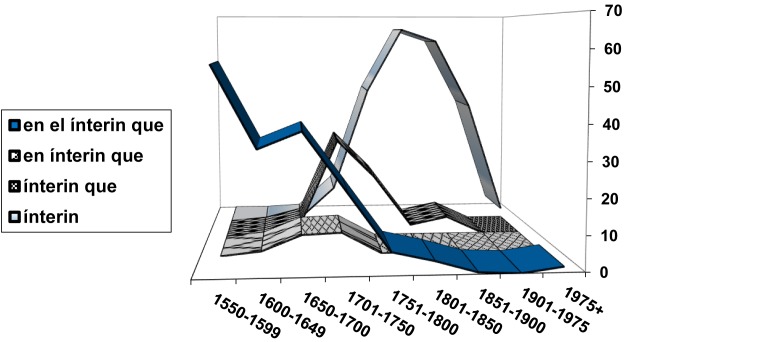
Evolution of relational uses of *ínterin* “while” [*lo acabaremos (en (el)) ínterin (que) los demás llegan* “we will finish it (in (the)) meantime the others arrive”]: Relative frequency percentages for each construction within each period. Data from CORDE.

Possibly, the best way to reach valid generalizations in research on linguistic diffusion is through trial and error. The process is thus fraught with error, but nevertheless worth the effort: the alternative would be to remain content with current remarks of diffusion, such as the increasing frequency of grammaticalized elements and constructions or the (alleged) universality of S-curve trajectories in cases of competing minimal pairs, which, as we expect to have illustrated, fall short of accounting for all the interesting ups and downs observed during propagation, and are in addition neither well suited to describe the final phases (regressive or otherwise) of trajectories, nor allow us to correlate types of change and modes of diffusion^25^. Furthermore, researchers of S-curve effects, despite typically attributing its peculiar sinuosity to social interaction between speakers, do not seem particularly inclined to a more detailed exploration of how the diffusion of individual changes manifests in distinct variational dimensions [as described e.g., by Koch and Oesterreicher (1990/2011)], or in how much they can be attributed to concrete textual traditions. The relentless regularity of the S-curve has a distinct appeal in its elegant simplicity and uniformity, but, for many historians of language, it also arouses the desire to transcend its monotony in search of the unstable, brittle heterogeneity that is in all probability as intrinsic to the diffusion of linguistic changes as to all other social activities engaged in by human beings.

## Author Contributions

The author confirms being the sole contributor of this work and has approved it for publication.

## Conflict of Interest Statement

The author declares that the research was conducted in the absence of any commercial or financial relationships that could be construed as a potential conflict of interest.

## Footnotes

1 https://ahle.webnode.es/

2 Cano (1995: 324) already noted the preponderance of papers dedicated to historical morphosyntax in the proceedings of the first two of these conferences, a dominance which has been maintained and indeed increased in subsequent proceedings. According to Concepción Company Company (2017), in the last nine proceedings up until the present, of the three major areas of study, morphosyntax was the subject of 29% of contributions (437/1514), whilst the following two, Lexicology and semantics, on one hand, and Historiography and history of the language, on the other, received far less attention: 22% (339/1514) and 19% (286/1514) publications, respectively.

3 www.rae.es

4 Concerning these topics, cf. Lucía (2003) and more recently, Garachana and Artigas (2012), Kabatek (2012, 2016), Lleal (2013), Octavio de Toledo y Huerta (2016a), or Octavio de Toledo y Huerta and Rodríguez Molina (2017). However, these problems occur to a much lesser degree in CORDE than in other competing corpora. This applies particularly to Mark Davies’ *Corpus del Español*, which offers clear benefits in terms of searchability, but is far less complete and includes texts of a (much) lower philological quality. For a critical comparison between CORDE and the *Corpus del Español*, cf. Davies (2009) and Rojo (2010); also of interest for an analysis of certain specific phenomena on both corpora are Nieuwenhuijsen (2009) and García Salido and Vázquez Rozas (2012). Concerning the general characteristics of CORDE, cf. also Sánchez and Domínguez (2007) and Rojo (2012), and for its use as a tool for syntactical analysis, cf. e.g., Sánchez Lancis (2009); Buenafuentes and Sánchez Lancis (2012), and Octavio de Toledo y Huerta (2016a).

5 Another 6 studies (17%) use Davies’ *Corpus del Español*, half of which (3/6) also use CORDE. There are 4 studies (11%) based on CODEA and 2 (5.5%) which rely on *Biblia Medieval* (one of these also uses CODEA). Only two studies use the holochronic methodology of selecting only two or three works per century, in one case this approach is supported by CORDE. Of those studies completed without recourse to an electronic corpus, the vast majority (6/9) are exclusively focussed on medieval Spanish, a fact which shows how studies which, exceptionally, do not use this type of corpus tend to adhere to the most traditional methods of data collection and are concerned with the language before the 1500’s. For the purposes of comparison with Table 1, the majority of contributions concerning morphosyntax at IX CIHLE are holochronic studies (17/36, 53%): these are followed by the studies focussing on the Middle Ages (14/36, 53%) and the remaining few are, as ever, those dedicated to Classical Spanish (3/36, 8%) and Modern Spanish (2/36, 6%). This confirms the findings concerning recent trends as shown on Table 1.

6 For further criticism of the “darker” aspects in conducting research with CORDE, I refer the reader to Octavio de Toledo y Huerta (2014a, 2016a), Fernández Alcaide et al. (2016), or Octavio de Toledo y Huerta and Rodríguez Molina (2017).

7 Three hundred and sixty-seven cases were retrieved through an exhaustive search of CORDE; cf. Octavio de Toledo y Huerta (2016b).

8 Thus, this line reflects the fact that in the 1485–1524 period, for instance, only around 5% of the total tokens are found, whereas almost 45% of the tokens concentrate in the last described period, 1605–1660.

9 For an analysis of the syntactic behavior and other properties of “analytic” futures (and conditionals), cf. Castillo (2002), Concepción Company Company (2006), Girón (2007); Bouzouita (2011), Octavio de Toledo y Huerta (2015a), and Batllori (2016).

10 For a more complete list of digital libraries available on the web, cf. Kabatek (2016: 15–16).

11 This digital portal often contains all those ancient books from an individual library that its librarians have decided to scan based on the book’s accessibility, state of preservation, date or place of publication, adscription to a certain collection or series, material value, and rarity, etc., without particular reference to the “literary” quality of these works. This clearly results in a much more diverse selection than usual in standard, closed diachronic corpora. For an analysis of the effects of esthetic prejudice on the preference for certain periods and works, which in turn influences editorial practice and thus the set of texts available in a philologically reliable form, cf. Pons (2006) and Montaner (2011). The main characteristics of a corpus that is authentically representative as suited to the needs of historical linguistics have been described by Kabatek (2013).

12 i.e., Characteristic of the eastern half of the Iberian Peninsula.

13 As an reviewer rightly points out, empirical research conducted directly on Google and similar platforms raises issues concerning the representativity of the data obtained, particularly when searching for competing variants. I address some such issues in Octavio de Toledo y Huerta (2016c), as do other contributions in that special number.

14 A parallel ongoing discussion concerns the universality of the Constant Rate Hypothesis (CRH), equally introduced by Kroch (1989), which predicts that syntactic change takes place at the same pace across different contexts: cf. now Kauhanen and Walkden (2018) for a refined version of the CRH.

15 “The expansion in the frequency of use of *haber* + PP in the period 1425–1524 was completely dependent on the expansion of *haber* + PP to new syntactic contexts. The relevance of this result lies in the fact that it permits the formulation of a hypothesis about the causes for the substitution of *ser* + PP by *haber* + PP in Spanish: our analysis suggests that the expansion of *haber* + PP into contexts of use where *ser* was previously used was caused by the syntactic factor of reflexivity” (Rosemeyer, 2016: 499 [my translation, ÁOdT]).

16 Of course, a slow inception of the curve before 1450 and a slow tapering-off after 1650 can be reasonably assumed: my focus here is on the rather unexpected quick-slow-quick pace in the central stages of the diffusion. The possibility of a difference between lexical expansion and other types of diffusion was already suggested by Denison (2003).

17 Alternatively, the first curve could correspond to the type that, instead of a tendency to generalization in the final stages, leads to “reasonably stable variation with the variants fluctuating around a mean percentage value” (Blythe and Croft, 2012: 280), a configuration which these authors subsume into the general category of the S-curve. Note that, consequently, the form taken by the progression of the final generalization, should there be any, is irrelevant to the consideration of a trajectory as an S-curve.

18 See the project’s web page at: http://gradiadiacronia.wixsite.com/gradia.

19 Incidentally, as indicated by Nevalainen (2015), in contrast to the phase of expansion in which the presence of S-curves is expected, their presence in the recessive phase requires additional explanation from a sociolinguistic point of view: “If the outcome is expected (with the benefit of hindsight, for example), the diffusion of linguistic change along an S-shaped curve does not necessarily call for an explanation, but a change reversal normally does.”

20 I deliberately leave aside those phenomena that Concepción Company Company (2017) dubs “continuous changes,” that is, those that do not display noticeable frequency alternations throughout their evolution. For other objections to the alleged universality of the S-curve as regards the diffusion of linguistic changes, cf. Denison (2003) or Winter-Froemel (2014).

21 On the theoretical necessity of doing so, cf. Fernández-Ordóñez (2011) for diastratic Eberenz (2009) or Kabatek (2012) for diaphasic considerations.

22 Suffice it to quote the much missed Wulf Oesterreicher: “questions about innovation strategies and so-called paths of grammaticalization should *always* be followed by questions concerning the discursive avenues of diffusion and successive adoption of these innovations by speakers” [Oesterreicher, 2006: 146 (original emphasis; my translation, ÁOdT)].

23 Interest in the interaction between frequency and the preference of individual phenomena for certain (groups of) texts and textual forms is breaking through in historical linguistics from several different methodological standpoints [cf. e.g., Szmrecsanyi, 2016 or Simonenko et al. (2018)]. The *discourse traditions* (or DT) framework (as expounded e.g., in Kabatek, 2017) offers, however, a useful specific set of theoretical notions and insights with which to track historical routes of diffusion across texts.

24 I expand on this proposal in more detail in Octavio de Toledo y Huerta (2016a).

25 In other words, “S-curves provide only a very coarse-grained description of the spreading of linguistic innovations in a population,” but S-curves themselves “can be used to discriminate between different mechanistic descriptions and to quantify the importance of different factors known to act on language change” (Ghanbarnejad et al., 2014: 8b).

## References

[B1] Academia Mexicana de la Lengua (2014). *Corpus Diacrónico y Diatópico del Español de América*. Available at: http://www.cordiam.org/

[B2] Andrés Enrique-Arias (2004). *Corpus Biblia Medieval.* Available at: http://www.bibliamedieval.es/

[B3] Anipa K. (2000). A study of the analytic future / conditional in Golden-Age Spanish. *Bull. Hisp. Stud.* 77 325–338.

[B4] Arnold T. Tilton L. (2015). *Humanities Data in R: Exploring Networks, Geospatial Data, Images, and Text.* Cham: Springer 10.3828/bhs.77.3.325

[B5] Batllori M. (2016). “El valor modal de *haber* en los futuros y condicionales analíticos,” in *En Torno a Haber: Construcciones, Usos y Variació*n Desde el Latín Hasta la Actualidad, eds de Benito Moreno C. Octavio de Toledo Á. (Franfurt: Peter Lang), 33–78. 10.1007/978-3-319-20702-5

[B6] Batllori M. Hernanz M. L. (2015). “Sentential focus and polarity: asymmetries between Spanish and Catalan,” in *Syntax Over Time: Lexical, Morphological and Information-Structural Interactions*, eds Biberauer T. Walkden G. (Oxford: Oxford University Press), 280–298.

[B7] Bernárdez E. (1994). “Can catastrophe theory provide adequate explanations for linguistic change? An application to syntactic change in English,” in *English Historical Linguistics 1992*, eds Fernández F. Fuster M. Calvo J. J. (Amsterdam: John Benjamins), 17–27.

[B8] Bivand R. S. Pebesma E. Gómez Rubio V. (2013). *Applied Spatial Data Analysis with R.* New York, NY: Springer 10.1007/978-1-4614-7618-4

[B9] Blythe R. Croft W. (2012). S-curves and the mechanisms of propagation in language change. *Language* 88 269–304. 10.1353/lan.2012.0027

[B10] Bouzouita M. (2011). “Future constructions in Medieval Spanish: mesoclisis uncovered,” in *The Dynamics of Lexical Interfaces*, eds Kempson R. Gregoromichelaki E. Howes C. (Stanford: CSLI Publications), 91–132.

[B11] Breban T. (2014). What is secondary grammaticalization? Trying to see the wood for the trees in a confusion of interpretations. *Folia Linguist.* 48 469–502. 10.1515/flin.2014.016

[B12] Buenafuentes C. Sánchez Lancis C. (2012). “Procesos de gramaticalización y lexicalización a la luz de los corpus académicos,” in *Cum Corde et in Nova Grammatica: Estudios Ofrecidos a Guillermo Rojo*, eds Jiménez Juliá T. López Meirama B. Vázquez Rozas V. Veiga Rodríguez A. (Santiago de Compostela: Universidade de Santiago de Compostela), 153–165.

[B13] Bustos G. E. Moreno B. J. (1992). “La asimetría *hemos* / *habéis*,” in *Actas del II Congreso Internacional de Historia de la Lengua Española*, Vol. I, eds Ariza M. Cano R. Mendoza J. M. A. Narbona Y. A. (Madrid: Pabellón de España), 307–321.

[B14] Bybee (2011). “Usage-based theory and grammaticalization,” in *The Oxford Handbook of Grammaticalization*, eds Narrog H. Heine B. (Oxford: Oxford University Press), 69–78.

[B15] Cano A. R. (1991). Perspectivas de la sintaxis histórica española. *Anu. Let.* 29 53–81.

[B16] Cano A. R. (1995). Problemas metodológicos en sintaxis histórica española. *Rev. Esp. Lingüíst.* 25 323–346.

[B17] Cano A. R. (2000). Oración compleja y estructura del discurso: nuevos desarrollos en sintaxis histórica del español. *Rev. Investig. Lingüíst.* 3 95–122.

[B18] Castillo L. M. (2002). “Distribución de las formas analíticas y sintéticas de futuro y condicional en español medieval,” in *Actas del V Congreso Internacional de Historia de la Lengua Española*, Vol. I, eds Echenique M. T. Sánchez Méndez J. (Madrid: Gredos), 541–549.

[B19] Concepción Company Company (2005). Una paradoja de la lingüística histórica romance: el florecimiento de la sintaxis histórica románica. *Corónica* 34 144–163. 10.1353/cor.2005.0039

[B20] Concepción Company Company (2017). “El siglo XIX en la periodización sintáctica de la lengua española,” in *Herencia e Innovació*n en el Español del Siglo XIX, eds Carpi E. García Jiménez R. (Pisa: Pisa University Press), 75–101.

[B21] Concepción Company Company (ed.) (2006). “Tiempos de formación romance II. Los futuros y condicionales,” in *Sintaxis Histó*rica de la Lengua Española, Vol. I, (Mexico: UNAM), 349–422.

[B22] Coseriu E. (1983). Linguistic change does not exist. *Linguist. Nuova Antica* 1 51–63.

[B23] Davies M. (2002). *Corpus del Español.* Available at: http://www.corpusdelespanol.org

[B24] Davies M. (2009). “Creating useful historical corpora: a comparison of CORDE, the *Corpus del Español*, and the *Corpus do Português*,” in *Diacroní*a de las Lenguas Iberorromances: Nuevas Perspectivas Desde la Lingüística de Corpus, ed. Enrique Arias A. (Madrid: Iberoamericana Vervuert), 137–166.

[B25] De Smet H. (2012). The course of actualization. *Language* 88 601–633. 10.1353/lan.2012.0056

[B26] De Smet H. (2013). How gradual change progresses: the interaction between convention and innovation. *Lang. Var. Change* 28 83–102. 10.1017/S0954394515000186

[B27] De Smet H. D’hoedt F. Fonteyn L. Van Goethem K. (2018). The changing functions of competing forms: attraction and differentiation. *Cogn. Linguist.* 29 197–234. 10.1515/cog-2016-0025

[B28] De Smet H. Fischer O. (2017). “The role of analogy in language change: supporting constructions,” in *The Changing English Language: Psycholinguistic Perspectives*, ed. Hundt M. (Cambridge: Cambridge University Press), 240–268. 10.1017/9781316091746.011

[B29] Denison D. (2003). “Logistic and simplistic S-curves,” in *Motives for Language Change*, ed. Hickey R. (Cambridge: Cambridge University Press), 54–70. 10.1017/CBO9780511486937.005

[B30] Eberenz R. (2009). La periodización de la historia morfosintáctica del español: propuestas y aportaciones recientes. *Cah. Étud. Hispaniques Méd.* 32 181–201. 10.3406/cehm.2009.2072

[B31] Feltgen Q. Fagard B. Nadal J. P. (2017). Frequency patterns of semantic change: corpus-based evidence of a near-critical dynamics in language change. *R. Soc. Open Sci.* 4:170830. 10.1098/rsos.170830 29291074 PMC5717648

[B32] Fernández Alcaide M. Leal Abad E. Octavio de Toledo Á. S. (2016). “*El mal considerado siglo nuestro*: problemas poco atendidos y fenómenos poco estudiados en el español del siglo XVII,” in *En la Estela del Quijote: Cambio Lingü*ístico, Normas y Tradiciones Discursivas en el Siglo XVII, eds Fernández Alcaide M. Leal E. Octavio de Toledo Á. (Frankfurt: Peter Lang), 9–44.

[B33] Fernández-Ordóñez I. (2011). *La Lengua de Castilla y la Formación del Español.* Madrid: Real Academia Española.

[B34] Garachana C. M. (2011). “Perífrasis sinónimas. ¿Gramaticalizaciones idénticas? Más retos para la teoría de la gramaticalización,” in *Sintaxis y Aná*lisis del Discurso Hablado en Español: Homenaje a Antonio Narbona, Vol. II, eds de Bustos J. J. Cano R. Méndez E. (Sevilla: Universidad de Sevilla), 779–798.

[B35] Garachana C. M. (2016). “Redundancias gramaticales en la expresión de la modalidad deóntica. La perífrasis *haber que + infinitivo* en la historia del español,” in *En Torno a Haber: Construcciones, Usos y Variació*n Desde el Latín Hasta la Actualidad, eds de Benito C. Octavio de Toledo Á. (Frankfurt: Peter Lang), 327–356.

[B36] Garachana C. M. Artigas E. (2012). Corpus digitalizados y palabras gramaticales. *Scriptum Digit.* 1 37–65.

[B37] Garachana C. M. Hernández Díaz A. (2017). “La reestructuración del sistema perifrástico en el español decimonónico. El caso de *haber de* / *tener de* + infinitivo, *haber que* / *tener que* + infinitivo,” in *Herencia e Innovació*n en el Español del Siglo XIX, eds Carpi E. García Jiménez R. (Pisa: Pisa University Press), 127–146.

[B38] García de Paredes M. E. (2011). “*Si yo fuera estado allí, no fuera pasado eso*. Pervivencia de un aparente arcaísmo en la lengua de Internet,” in *Sintaxis y Aná*lisis del Discurso Hablado en Español: Homenaje a Antonio Narbona, Vol. II, ed. de Bustos Tovar J. J. (Sevilla: Universidad de Sevilla), 1009–1032.

[B39] García Salido M. Vázquez Rozas V. (2012). Los corpus diacrónicos como instrumento para el estudio del origen y distribución de la concordancia de objeto en español. *Scriptum Digit.* 1 67–84.

[B40] Ghanbarnejad F. Gerlach M. Miotto J. M. Altmann E. G. (2014). Extracting information from S-curves of language change. *J. R. Soc. Interface* 11:20141044. 10.1098/rsif.2014.1044 25339692 PMC4223929

[B41] Girón A. J. L. (2004). Gramaticalización y estado latente. *Dicenda* 22 71–88.

[B42] Girón A. J. L. (2005). Perspectivas de la lingüística histórica románica e hispánica. *Corónica* 34 176–189. 10.1353/cor.2005.0017

[B43] Girón A. J. L. (2007). “De nuevo sobre la gramaticalización del futuro analítico,” in *Ex Admiratione et Amicitia: Homenaje a Ramó*n Santiago, Vol. I, eds Delgado I. Puigvert A. (Madrid: Ediciones del Orto), 563–576.

[B44] Girón A. J. L. (ed.) (2006). “Un siglo de sintaxis histórica del español,” in *Filologí*a y Lingüística: Estudios Ofrecidos a Antonio Quilis, Vol. II, (Madrid: CSIC), 1745–1762.

[B45] Gries S. T. (2009). *Statistics for Linguistics with R: A Practical Introduction.* Berlin: De Gruyter 10.1515/9783110216042

[B46] Haspelmath M. (2004). “On directionality in language change with particular reference to grammaticalization,” in *Up and Down the Cline — The Nature of Grammaticalization*, eds Fischer O. Norde M. Perridon H. (Amsterdam: John Benjamins), 17–44. 10.1075/tsl.59.03has

[B47] Heine B. (1992). Grammaticalization chains. *Stud. Lang.* 16 335–368. 10.1075/sl.16.2.05hei

[B48] Herrero F. J. (2013). “*El que sea esto así, yo lo sé.* Aproximación histórica a las oraciones subordinadas sustantivas precedidas de artículo,” in *Literatura, Pasió*n Sagrada. Homenaje al Profesor Antonio García Berrio, eds González Alcázar C. F. Moreno F. Á. Villar J. F. (Madrid: Editorial Complutense), 445–462.

[B49] Himmelmann N. P. (2004). “Lexicalization and grammaticization: opposite or orthogonal?,” in *What Makes Grammaticalization? A Look Rom its Fringes and its Components*, eds Bisang W. Himmelmann N. P. Wiemer B. (Berlin: De Gruyter), 21–42.

[B50] Jaeger G. Rosenbach A. (2008). Priming and unidirectional language change. *Theor. Linguist.* 34 85–113.

[B51] Jenset G. B. McGillivray B. (2017). *Quantitative Historical Linguistics: A Corpus Framework.* Oxford: Oxford University Press 10.1093/oso/9780198718178.001.0001

[B52] Kabatek J. (2012). “Nuevos rumbos en la sintaxis histórica,” in *Actas del VIII Congreso Internacional de Historia de la Lengua Española*, Vol. I, ed. Montero Cartelle E. (Santiago de Compostela: Meubook), 77–100.

[B53] Kabatek J. (2013). ¿Es posible una lingüística histórica basada en un corpus representativo? *Iberoromania* 77 8–28. 10.1515/ibero-2013-0045

[B54] Kabatek J. (2015). “Warum die ‘zweite Historizität’ eben doch die zweite ist – von der Bedeutung von Diskurstraditionen für die Sprachbetrachtung,” in *Diskurse, Texte, Traditionen: Modelle und Fachkulturen in der Diskussion*, eds Lebsanft F. Schrott A. (Bonn: Bonn University Press), 49–62.

[B55] Kabatek J. (2016). “Un nuevo capítulo en la lingüística histórica iberrománica: el trabajo crítico con los corpus,” in *Lingüí*stica de Corpus y Lingüística Histórica Iberorrománica, ed. Kabatek J. (Berlin: De Gruyter), 1–17.

[B56] Kabatek J. (2017). *Lingüística Coseriana, Lingüística Histórica, Tradiciones Discursivas.* Madrid: Iberoamericana Vervuert.

[B57] Kauhanen H. Walkden G. (2018). Deriving the constant rate effect. *Nat. Lang. Linguist. Theory* 36 483–521. 10.1007/s11049-017-9380-1

[B58] Killie K. (2015). Secondary grammaticalization and the English adverbial *-ly* suffix. *Lang. Sci.* 47 199–214. 10.1016/j.langsci.2014.10.003

[B59] Koch P. Oesterreicher W. (1990/2011). *Gesprochene Sprache in der Romania: Französisch, Italienisch, Spanisch.* Berlín: De Gruyter 10.1515/9783111372914

[B60] Kroch A. (1989). Reflexes of grammar in patterns of language change. *Lang. Var. Change* 1 199–244. 10.1017/S0954394500000168

[B61] Lapesa M. R. (1984). “El uso de actualizadores con el infinitivo y la suboración sustantiva en español: diacronía y sentido,” in *Homenaje a Ana Marí*a Barrenechea, eds Schwartz L. Lerner I. (Madrid: Castalia), 65–89.

[B62] Levshina N. (2015). *How to Do Linguistics with R: Data Exploration and Statistical Analysis.* Amsterdam: John Benjamins 10.1075/z.195

[B63] Lleal G. C. (2013). Rigor metodológico e investigación filológica. *Scriptum Digit.* 2 107–121.

[B64] López García Á. (1996). Teoría de catástrofes y variación lingüística. *Rev. Esp. Lingüíst.* 26 15–42.

[B65] López García Á. (2011). “Formas de pensar la historia en español,” in *Sintaxis y Aná*lisis del Discurso Hablado en Español: Homenaje a Antonio Narbona, Vol. II, eds de Bustos J. J. Cano R. Méndez E. (Sevilla: Universidad de Sevilla), 637–652.

[B66] Lucía M. J. M. (2003). La informática humanística: notas volanderas en el ámbito hispánico. *Incipit* 23 91–114.

[B67] Mackenzie I. (2010). Refining the V2 hypothesis for old Spanish. *Bull. Hisp. Stud.* 87 379–396. 10.3828/bhs.2010.8

[B68] Mackenzie I. (2017). The rise and fall of proclisis in Old Spanish postprepositional infinitival clauses: a quantitative approach. *Bull. Hisp. Stud.* 94 127–146. 10.3828/bhs.2017.9

[B69] Malkiel Y. (1948). *Hispanic Alguien and Related Ormations: A Study of the Stratification of the Romance Lexicon in the Iberian Peninsula.* Berkeley: University of California Press.

[B70] Marco C. Marín R. (2015). “Origins and development of adjectival passives in Spanish: a corpus study,” in *New Perspectives on the Study of Ser and Estar*, eds Pérez-Jiménez I. Leonetti M. Gumiel-Molina S. (Amsterdam: John Benjamins), 239–266. 10.1075/ihll.5.09mar

[B71] Montaner F. A. (2011). Factores empíricos en la conformación del canon literario. *Stud. Aurea* 5 49–70. 10.5565/rev/studiaaurea.19

[B72] Morala J. R. (2002). “Nuevas tecnologías y recursos lexicográficos: *fuereño*,” in *Filologí*a en Internet, ed. Clavería G. (Barcelona: Universitat Autònoma de Barcelona), 45–53.

[B73] Nevalainen T. (2015). *Descriptive Adequacy of the S-Curve Model in Diachronic Studies of Language Change. VARIANT 16.* Available at: http://www.helsinki.fi/varieng/journal/volumes/16/nevalainen/

[B74] Nieuwenhuijsen D. (2009). “El rastreo del desarrollo de algunos pronombres personales en español: (im)posibilidades de los corpus diacrónicos digitales,” in *Diacroní*a de las Lenguas Iberorrománicas: Nuevas Aportaciones Desde la Lingüística de Corpus, ed. Enrique Arias A. (Madrid: Iberoamericana Vervuert), 365–384.

[B75] Norde M. (2012). “Lehmann’s parameters revisited,” in *Grammaticalization and Language Change: New Reflections*, eds Davidse K. Leuschner T. (Amsterdam: John Benjamins), 73–110. 10.1075/slcs.130.04nor

[B76] Octavio de Toledo y Huerta Á. S. (2007). “Un rasgo sintáctico del primer español moderno (ca. 1675-1825): las relaciones interoracionales con *í*nterin (*que*),” in *Cuatrocientos Años de la Lengua del Quijote: Estudios de Historiografía e Historia de la Lengua Española*, eds Fernández Alcaide M. López Serena A. (Sevilla: Universidad de Sevilla), 421–442.

[B77] Octavio de Toledo y Huerta Á. S. (2008). “Un nuevo esquema adversativo en el primer español moderno (ca. 1675-1825): la historia del nexo *sino es*,” in *Actas del VII Congreso Internacional de Historia de la Lengua Española*, Vol. I, eds Moreno de Alba J. G. Concepción Company Company (Madrid: Arco Libros), 877–907.

[B78] Octavio de Toledo y Huerta Á. S. (2014a). “Entre gramaticalización, estructura informativa y tradiciones discursivas: algo más sobre nada,” in *Procesos de Gramaticalizació*n en la Historia del Español, eds Girón Alconchel J. L. Sáez Rivera D. M. (Madrid: Iberoamericana Vervuert), 263–319.

[B79] Octavio de Toledo y Huerta Á. S. (2014b). Espejismo de la frecuencia creciente: gramaticalización y difusión del artículo ante oraciones sustantivas. *RILCE* 30 916–958.

[B80] Octavio de Toledo y Huerta Á. S. (2015a). “Futuros que se miran el ombligo: mesoclisis y anteposición de formas no personales en la historia del español,” in *El Orden de Palabras en la Historia del Españ*ol y Otras Lenguas Iberorromances, eds Castillo Lluch M. López Izquierdo M. (Madrid: Visor Libros), 141–233.

[B81] Octavio de Toledo y Huerta Á. S. (2015b). “La oculta vida dialectal de *bajo*+SN,” in *Actas del IX Congreso Internacional de Historia de la Lengua Españ*ola, Vol. II, ed. García Martín J. M. (Madrid: Iberoamericana Vervuert), 1841–1858.

[B82] Octavio de Toledo y Huerta Á. S. (2016a). “El aprovechamiento del CORDE para el estudio sintáctico del primer español moderno (ca. 1675-1825),” in *Lingüí*stica de Corpus y Lingüística Histórica Iberorrománica, ed. Kabatek J. (Berlin: De Gruyter), 29–54.

[B83] Octavio de Toledo y Huerta Á. S. (2016b). Enseñanzas del cambio fracasado: trayectoria y estela de una perífrasis fugaz (infinitivo + *tener*). *Cuad. Lingüíst. Col. México* 3 119–181.

[B84] Octavio de Toledo y Huerta Á. S. (2016c). Sin CORDE pero con red: algotras fuentes de datos. *Rev. Int. Lingüíst. Iberoam.* 28 19–48.

[B85] Octavio de Toledo y Huerta Á. S. (2018a). De un occidentalismo cortesano y una transfusión fallida: historia de *es(t)otro*. *Estud. Lingüíst. Esp.* 39 305–361.

[B86] Octavio de Toledo y Huerta Á. S. (2018b). “¿Tradiciones discursivas o *tradicionalidad*? ¿Gramaticalización o *sintactización*? Difusión y declive de las construcciones modales con infinitivo antepuesto,” in *Procesos de Textualizació*n y Gramaticalización en la Historia del Español, eds Girón J. L. Herrero F. J. Sáez D. M. (Madrid: Iberoamericana Vervuert), 79–134. 10.31819/9783954876938-004

[B87] Octavio de Toledo y Huerta Á. S. Rodríguez Molina J. (2017). La necesaria distinción entre texto y testimonio: el CORDE y los criterios de fiabilidad lingüística. *Scriptum Digit.* 6 5–68.

[B88] Oesterreicher W. (2006). “La historicidad del lenguaje: variación, diversidad y cambio lingüístico,” in *Actas del VI Congreso Internacional de Historia de la Lengua Española*, Vol. I, eds Jesús de Bustos J. Luis Girón J. (Madrid: Arco Libros), 137–158.

[B89] Pato E. (2009). “Notas aclaratorias sobre la historia del indefinido *alguien*: una aplicación directa del uso de corpus diacrónicos,” in *Diacroní*a de las Lenguas Iberorrománicas: Nuevas Aportaciones Desde la Lingüística de Corpus, ed. Enrique-Arias A. (Madrid: Iberoamericana Vervuert), 401–416.

[B90] Pons R. L. (2006). “Canon, edición de textos e historia de la lengua cuatrocentista,” in *Historia de la Lengua y Crí*tica Textual, ed. Lola Pons (Madrid: Iberoamericana Vervuert), 69–126.

[B91] Postma G. (2010). “The impact of failed changes,” in *Continuity and Change in Grammar*, eds Breitbarth A. Lucas C. Watts S. Willis D. (Amsterdam: John Benjamins), 269–302. 10.1075/la.159.13pos

[B92] Real Academia Española (2000a). *Corpus de Referencia del Español Actual*. Available at: http://www.rae.es/

[B93] Real Academia Española (2000b). *Corpus Diacrónico del Español.* Available at: http://www.rae.es/

[B94] Real Academia Española (2014a). *Corpus del Español del Siglo XXI.* Available at: http://web.frl.es/CORPES/view/inicioExterno.view

[B95] Real Academia Española (2014b). *Corpus del Nuevo Diccionario Histórico del Español.* Available at: http://www.rae.es/

[B96] Rodríguez Molina J. (2004). Difusión léxica, cambio semántico y gramaticalización: el caso de *haber* + participio en español antiguo. *Rev. Filol. Esp.* 84 169–209. 10.3989/rfe.2004.v84.i1.102 13012110

[B97] Rodríguez Molina J. (2012). “La reducción fonética *habemos cantado>hemos cantado* en español antiguo: nuevos datos y nuevas hipótesis,” in *Estudios de Filologí*a y Lingüística Españolas: Nuevas Voces en la Disciplina, eds Pato E. Rodríguez Molina J. (Bern: Peter Lang), 167–233.

[B98] Rojo G. (2010). Sobre codificación y explotación de corpus textuales: otra comparación del *Corpus del Español* con el CORDE y el CREA. *Lingüística* 24 11–50.

[B99] Rojo G. (2012). “El papel de los corpus en el estudio de la historia del español,” in *Actas del IX Congreso Internacional de Historia de la Lengua Española*, Vol. I, ed. Montero Cartelle E. (Santiago de Compostela: Meubook), 433–444.

[B100] Rosemeyer M. (2014). *Auxiliary Election in Spanish: Gradience, Gradualness, and Conservation.* Amsterdam: John Benjamins 10.1075/slcs.155

[B101] Rosemeyer M. (2016). “Gradientes semánticos y sintácticos en la historia de la selección de auxiliares en español,” in *En Torno a Haber: Construcciones, Usos y Variació*n Desde el Latín Hasta la Actualidad, eds de Benito Moreno C. Octavio de Toledo Á. S. (Frankfurt: Peter Lang), 469–502.

[B102] Rosemeyer M. Schwenter S. A. (2017). Entrenchment and persistence in language change: the Spanish past subjunctive. *Corpus Linguist. Linguist. Theory* 15 10.1515/cllt-2016-0047 [Epub ahead of print].

[B103] Sánchez S. M. Domínguez C. C. (2007). El banco de datos de la Real Academia Española: CREA y CORDE. *Per Abbat* 2 137–146.

[B104] Sánchez Lancis C. (2009). Corpus diacrónicos y periodización del español. *Cah. Étud. Hispaniques Méd.* 32 159–180. 10.3406/cehm.2009.2071

[B105] Sánchez Marco C. (2012). *Tracing the Development of Spanish Participation Constructions: An Empirical Study of Semantic Change.* Doctoral dissertation, Universitat Pompeu Fabra, Barcelona.

[B106] Sánchez-Prieto Borja P. (2015). *Corpus de Documentos Españoles Anteriores A 1800.* Available at: http://corpuscodea.es/

[B107] Schmid H. J. (2016). “A framework for understanding linguistic entrenchment and its psychological foundations,” in *Entrenchment and the Psychology of Language Learning*, ed. Schmid H.-J. (Berlin: De Gruyter), 9–35.

[B108] Simonenko A. Crabbé B. Prévost S. (2018). Text form and grammatical changes in Medieval French: a treebank-based diachronic study. *Diachronica* 35 393–428. 10.1075/dia.00008.sim

[B109] Sitaridou I. (2011). Word order and information structure in Old Spanish. *Catalan J. Linguist.* 10 159–184. 10.5565/rev/catjl.36

[B110] Sitaridou I. Eide K. G. (2014). “Contrastivity and information structure in the Old Ibero-Romance languages,” in *Information Structure and Word Order in Old Germanic and Old Romance*, eds Bech K. Eide K. G. (Amsterdam: John Benjamins), 377–412.

[B111] Szmrecsanyi B. (2005). Language users as creatures of habit: a corpus-based analysis of persistence in spoken English. *Corpus Linguist. Linguist. Theory* 1 113–150. 10.1515/cllt.2005.1.1.113

[B112] Szmrecsanyi B. (2016). About text frequencies in historical linguistics: disentangling environmental and grammatical change. *Corpus Linguist. Linguist. Theory* 12 153–171. 10.1515/cllt-2015-0068

[B113] Torres C. R. (2009). “Las nominalizaciones de infinitivo,” in *Sintaxis Histó*rica de la Lengua Española, Vol. II, ed. Concepción Company Company (Mexico: UNAM), 1673–1738.

[B114] Torres C. R. (2015). “Gradual loss of analyzability: diachronic priming effects,” in *Variation in Language: System- and Usage-Based Approaches*, eds Adli A. Kaufmann G. García M. (Berlin: De Gruyter), 267–289.

[B115] Universitat d’Alacant (1999). *Biblioteca Virtual Miguel de Cervantes.* Available at: http://www.cervantesvirtual.com

[B116] Varga E. (2017). *Verbstellungsmuster im Altfranzösischen. Ein Beitrag zur Historischen Syntaxforschung aus Diskurstraditioneller Perspektive.* Berlin: De Gruyter 10.1515/9783110536591

[B117] Winter-Froemel E. (2014). What does it mean to explain language change? Usage-based perspectives on causal and intentional approaches to linguistic diachrony, or: on S-curves, invisible hands, and speaker creativity. *Energeia* 5 123–142.

[B118] Winter-Froemel E. López Serena A. Octavio de Toledo Á. S. Frank-Job B. (eds) (2015). “Diskurstraditionen, diskurstraditionelles und einzelsprachliches im sprachwandel: zur einleitung,” in *Diskurstraditionelles und Einzelsprachliches im Sprachwandel*, (Tübingen: Narr), 1–27.

